# Biochemical Requirements for Two Dicer-Like Activities from Wheat Germ

**DOI:** 10.1371/journal.pone.0116736

**Published:** 2015-01-23

**Authors:** Padubidri V. Shivaprasad, Thomas Hohn, Rashid Akbergenov

**Affiliations:** 1 National Centre for Biological Sciences, GKVK Campus, Bangalore, India; 2 Department of Plant Physiology, Botanical Institute, University of Basel, Basel, Switzerland; 3 Institute for Medical Microbiology, University of Zurich, Zurich, Switzerland; CNRS UMR7622 & University Paris 6 Pierre-et-Marie-Curie, FRANCE

## Abstract

RNA silencing pathways were first discovered in plants. Through genetic analysis, it has been established that the key silencing components called Dicer-like (DCL) genes have been shown to cooperatively process RNA substrates of multiple origin into distinct 21, 22 and 24 nt small RNAs. However, only few detailed biochemical analysis of the corresponding complexes has been carried out in plants, mainly due to the large unstable complexes that are hard to obtain or reconstitute in heterologous systems. Reconstitution of activity needs thorough understanding of all protein partners in the complex, something that is still an ongoing process in plant systems. Here, we use biochemical analysis to uncover properties of two previously identified native dicer-like activities from wheat germ. We find that standard wheat germ extract contains Dicer-like enzymes that convert double-stranded RNA (dsRNA) into two classes of small interfering RNAs of 21 and 24 nt in size. The 21 nt dicing activity, likely an siRNA producing complex known as DCL4, is 950 kDa-1.2 mDa in size and is highly unstable during purification processes but has a rather vast range for activity. On the contrary, the 24 nt dicing complex, likely the DCL3 activity, is relatively stable and comparatively smaller in size, but has stricter conditions for effective processing of dsRNA substrates. While both activities could process completely complementary dsRNA albeit with varying abilities, we show that DCL3-like 24 nt producing activity is equally good in processing incompletely complementary RNAs.

## Introduction

Eukaryotes possess an efficient system of gene regulation through the production of small RNA (sRNA) called RNA silencing. The 21–24 nt sRNAs are either generated from partial complimentary precursor RNAs or a completely complimentary double-stranded RNAs (dsRNA) of multiple and often exogenous origin. The small RNAs which are produced from partial complimentary precursors are either involved in mRNA degradation or in inhibition of mRNA translation are termed as micro (mi) RNAs with important roles in development and disease. The small RNAs of 21–24 nt produced from perfect complimentary RNA molecules have much wider roles, such as resistance against pathogens, controlling transposons, development and heterochromatin formation [1–4].

Small RNAs are processed from RNA substrates through the action of Dicer-like (DCL) proteins in plants. They can process RNAs based on their origin and structure into 21–24 nts. Once small RNAs are formed, they have the opportunity to make complexes with Argonautes (AGO) and this ribo-nucleoprotein complex is responsible for targeting RNAs that have sequence complementarity with small RNAs. Such a targeting can result in mRNA degradation or translational inhibition. This suppression of RNAs acts as a natural defense mechanism evolved to protect eukaryotic genomes against invasive nucleic acids such as viruses, transposable elements and transgenes. RNA silencing has emerged as a powerful tool with a wide range of applications in functional genomics and genetic engineering.

Dicers and DCLs are large (∼200 kDa), multidomain proteins that contain a putative RNA helicase domain, PAZ (Piwi/Argonaute/Zwille) domain, two tandem ribonuclease III (RNase III) domains and one or two dsRNA-binding domains [5]. In humans, Dicer preferentially processes siRNAs from the ends of dsRNAs [6] and a single Dicer is involved in both miRNA and siRNA production [7]. However, in other organisms these functions are mediated by two Dicers as in *Drosophila* and four or more Dicer-like (DCL) proteins in *Arabidopsis* and other plants [8,9].

All the four Arabidopsis DCLs have specialized functions [10,11]. DCL1, previously known as Carpel Factory (CAF) and Short Integuments1 (SIN1) is required for the production of miRNAs and trans-acting siRNAs (ta-siRNAs) [12,13]. DCL2 is involved in producing natural-antisense transcript siRNA (nat-siRNA) [14] and siRNAs against viruses [15–18]. DCL3 produces repeat-associated siRNA (ra-siRNA) that are involved in DNA methylation and histone modifications in heterochromatin region [18]. DCL4 is implicated in the production of transacting (ta)-siRNAs (which are involved in growth and development-related functions) and siRNAs from invading viruses and endogenous inverted repeats [12,19–22]. In more complex genomes such as monocots, DCLs can be 6–7 in number, appearing to be results of duplication of one or more core DCLs [5,23]. The additional DCLs present in monocots seem to be having special functions that have not been observed in *Arabidopsis* [24–28].

Plant DCLs are not known to act on dsRNA substrates alone. Few additional factors have been associated with DCL action on RNAs. In plants, such as *Arabidopsis*, few dsRNA-binding proteins are involved in RNA silencing and are known to act synergistically [29]. HYPONASTIC LEAVES (*Hyl1*) gene encodes a dsRNA-binding protein with two dsRNA-binding motifs, a putative nuclear localisation signal and a C-terminal repeat structure that may be involved in protein-protein interaction [30]. HYL1 functions in miRNA and ta-siRNA pathways as a partner of DCL1 and hyl1 mutants reflect phenotypes that are similar to other miRNA biogenesis mutants [31–38]. SERRATE is another nuclear factor required for miRNA biogenesis in plants. It is a C2H2 zinc finger protein [39–41] and has TOUGH as partner for its role in miRNA biogenesis [42]. A set of five double-stranded RNA binding proteins similar to HYL1 (DRB1) are essential parts of other DCL complexes in *Arabidopsis*, functionally equivalent to dsRNA binding proteins in animals [43,44].

One strand of siRNA is incorporated in an RNA-induced silencing complex (RISC) which guides ATP-dependent cleavage of homologous transcripts at a position corresponding to the nucleotides 10 and 11 of the siRNA. An important component of RISC complex is the AGO protein. AGOs are highly basic ∼100 kDa proteins and have been identified to be involved in RNA silencing through mutant screens. They have the characteristic N-terminal PAZ domain and a C-terminal PIWI domain. AGO1 functions as ‘slicer’ in *Arabidopsis*. AGO4 functions in different small RNA pathways related to DNA methylation and epigenetic regulation. AGO7/ZIPPY function in developmental timing and AGO10 (Zwille) resembles AGO1 in its function. Other plant AGOs have similar key roles during plant development and genome regulation (reviewed in [45]).

Examining the poplar and rice genomes revealed that they contain five and six Dicer-like genes, respectively [46]. Analysis of DCLs suggests that plants require a basic set of four Dicer types which were present before the divergence of mono- and dicotyledonous plants, but after the divergence of plants from green algae. Wheat and barley seem to have five DCLs, with DCL3 having two forms namely, DCL3a and DCL3b [46], functions of which are being actively investigated [47–49].

Although indirect effects of temperature, bivalent cations and substrates for DCL activity has been established [50–54], there is scarce information regarding nature and biochemical requirements of these activities. Experiments in *Arabidopsis* using DCL activities derived from flowers or immature floral buds were not successful mainly due to the unstable nature of protein complexes (unpublished results from the lab). However, *Arabidopsis* cell culture derived cells were good enough to isolate native DCL1 and DCL3 complexes [55], although the study was focused on identifying multiple silencing activities rather than to understand requirements for DCL action on RNA substrates. *Arabidopsis* seedling-derived crude extracts exhibited DCL4 activities and importance of DRB4 for their action [56]. However this work involved crude extracts where involvement from other DCL activities could not be established. *Arabidopsis* crude extracts were also used by the same group to show difference in the specificity for substrates by DCL3 (that prefers short substrate) and DCL 4 (prefers long substrate) [57]. At the same time, reconstitution of plant DCL activities in alternate systems, unlike in animal dicers, has not been very successful except in few cases. *In vitro* reconstituted pri-miRNA processing reactions using recombinant DCL1 indicated that DCL1 alone is able to release miRNA:miRNA* duplex, but the processing was inaccurate [32]. An extended work with recombinant DCL1 and SE expressed in insect cells reported that presence of SE improved accuracy of miRNA processing [58].

Extremely valuable information using native DCL complexes comes from limited studies in plants using wheat germ as the source of native protein complexes. Tang et al. [59] showed that extracts of wheat germ exhibit many of the key features of RNA silencing in plants. Using wheat germ *in vitro* system, they showed that in plants ATP-dependent, DCL enzymes cleave dsRNA into small RNAs that have the structure of siRNAs. Tang et al. [59] also show that wheat RdRP activity can synthesize dsRNA using exogenous single-stranded RNA as a template without an exogenous primer, and also evidence for RISC activity in wheat germ.

Here we have developed an *in vitro* system wherein the individual complexes of wheat germ are further purified to have either a 21 nt processing activity (previously identified as siRNA processing complex, likely DCL4 activity by Tang et al.) and a 24 nt processing activity (likely DCL3 as this is the only DCL that is capable of generating 24 nt siRNAs efficiently). Each of these native complexes were subjected to *in vitro* assays to determine their biochemical requirements to process dsRNAs into small RNAs. Our results largely correlate with the genetic analysis done using *A. thaliana* model system, for example, temperature sensitivity of DCLs as well as inhibition of their action due to certain bivalent cations. This is one of the few biochemical attempts to understand cleaving abilities of few individual plant DCLs and the method holds promise for further characterization to understand mechanism of DCL action.

## Materials and Methods

### Preparation of wheat germ

Wheat germs were prepared according to protocol described in Madin et al. [60]. Briefly, wheat seeds were ground in a mill (Rotor Speed Mill model pulverisette 14, Fritsh, Germany), sieved through a 710- to 850-mm mesh. Embryos were selected with the solvent flotation method of Anderson et al. [61] by using a solvent containing cyclohexane and carbon tetrachloride (240:600, v/v). Damaged embryos and contaminants were discarded, and intact embryos were dried overnight in a fume hood.

### S23 extract preparation

The method used is a slight modification of the procedure described by Madin et al. [60]. Washed embryos were ground to a fine powder in liquid nitrogen. Five grams of the powder were added to 5 ml of 2 × buffer A (20 mM Tris-Ac, pH 7.6,100 mM potassium acetate (KAc), 5 mM magnesium acetate, 5 mM DTT). The mixture was briefly vortexed and then centrifuged at 30,000 × *g* for 30 min. The resulting supernatant was subjected to gel-filtration on a G-25 (fine) column, equilibrated with two volumes of buffer A. The void volume was collected and centrifuged at 30,000 × g for 10 min. Extracts were frozen until use.

### Buffer composition for gel filtrations and all other columns

Following buffers were used for column chromatography. Sephacryl S300 gel filtration buffer: 20 mM Tris-Acetate (pH 7.6), 90 mM KAc, 2 mM MgAc, 10 mM MET, 5% glycerol. Q-sepharose column buffer: 20mM Tris-HCl (pH7.5), 50 mM KCl, 4 mM MgCl2, 8 mM MET, 5% glycerol, gradient of KCl from 50 to 500mM. DCL4 was eluted at 220 mM of KCl and DCL3 at 260 mM.

### Ammonium sulphate precipitation

Ammonium sulphate (Sigma-Aldrich) was used at various concentrations to precipitate proteins. The precipitated proteins are desalted using desalting columns (PD-10, GE healthcare), and used for functional assays.

### Generation of dsRNA substrates

To produce 700 bp long dsRNA, two PCR products made on template of GFP sequence (pEGFP-1) were cloned into the base plasmid. The first PCR product was produced using primer pairs: 5’-TAATACGACTCACTATAGGGatggtgagcaagggcgaggagctg (contains T7 promoter directing production of sense GFP RNA is in caps) and 5’-AAGCTTttacttgtacagctcgtccatgccga (*Hin*dIII restriction site is in caps). The second PCR product was amplified using primers:5’-TAATACGACTCACTATAGGGttacttgtacagctcgtccatgccga (contains T7 promoter directing production of anti-sense GFP RNA) and 5’- AAGCTTatggtgagcaagggcgaggagctg (containing *Hin*dIII restriction site). From the resulting clones, sense and antisense RNAs were transcribed using T7 polymerase in the presence of P32 UTP after digesting with *Hin*dIII. Sense and antisense RNAs were annealed to produce 700 bp dsRNA. This labelled dsRNA substrate was used for all dsRNA processing assays except the ones presented for substrate specificities.

The following oligonucleotides were used to make dsRNA substrates of 60 bp versions: SENSE1 FOR (5’-TAATACGACTCACTATAGGGACTTACAACAGTACGAATGTTACAATCAGATTCATAGTTAACTGAGGCCCGCGCCC) and SENSE1:REV (5’-GGGCGCGGGC CTCAGTTAACTATGAATCTGATTGTAACATTCGTACTGTTGTAAGTCCCTATAGTGAGTCGTATTA) oligos were annealed together and then transcribed using T7 polymerase (Fermentas) in the presence of alpha P32 UTP to produce a 60 nt sense RNA.

Similarly, ANTISENSE1(cc)FOR (5’-TAATACGACTCACTATAGGGCGCGGGCCTCAGTTA ACTATGAATCTGATTGTAACATTCGTACTGTTGTAAGTCCC and ANTISENSE1(cc)REV (5’-GGGACTTACAACAGTACGAATGTTACAATCAGATTCATAGTTAACTGA GGCCCGCGCCCTATAGTGAGTCGTATTA) were annealed together and transcribed to make a substrate that is complementary to sense RNA (60 nt antisense RNA). These two RNAs were annealed together to make a 60 bp dsRNA (60-P). Oligos ANTISENSE2(LOOP)FOR (5’-TAATACGACTCACTATAGGGCGCGGGCCTCACTTAACTATGATTCTGATTGTAA GTTTCGTACTGTAGTAAGTCCC) and ANTISENSE2(LOOP)REV (5’-GGGACTTACTAC AGTACGAAACTTACAATCAGAATCATAGTTAAGTGAGGCCCGCGCCCTATAGTGAGTCGTATTAT) were annealed and then transcribed to make a 60 nt antisense RNA that when annealed with 60 nt sense RNA can make imperfectly complementary 60 bp dsRNA (60-IMP).

### Analysis of dsRNA processing

For analysis of dsRNA processing, 5 nM internally -32P-UTP labeled dsRNA was incubated in a 10 μL reaction containing 5μL of wheat germ extract, 100 μM GTP, 500 μM ATP, 10 mM creatine phosphate, 10 μg/mL creatine phosphokinase, 5 mM DTT, and 0.1 U/μL RNasin (Promega) at 25°C for 3 h. Reactions were stopped by the addition of 2× proteinase K buffer (200 mM Tris-HCl at pH 7.5, 25 mM EDTA, 300 mMNaCl, 2% (w/v) sodium dodecyl sulfate) and deproteinized with ∼2 mg/mL proteinase K at 65°C for 15 min. Products were precipitated with 3 volumes cold ethanol and analyzed by electrophoresis in a 15% polyacrylamide gel. Radiolabelled size marker was prepared as discussed previously [62]. The blots were scanned using Typhoon FLA 9500 (GE healthcare).

## Results

### Detection of DCL activities from wheat germ extract and their size determination

**Figure 1:**
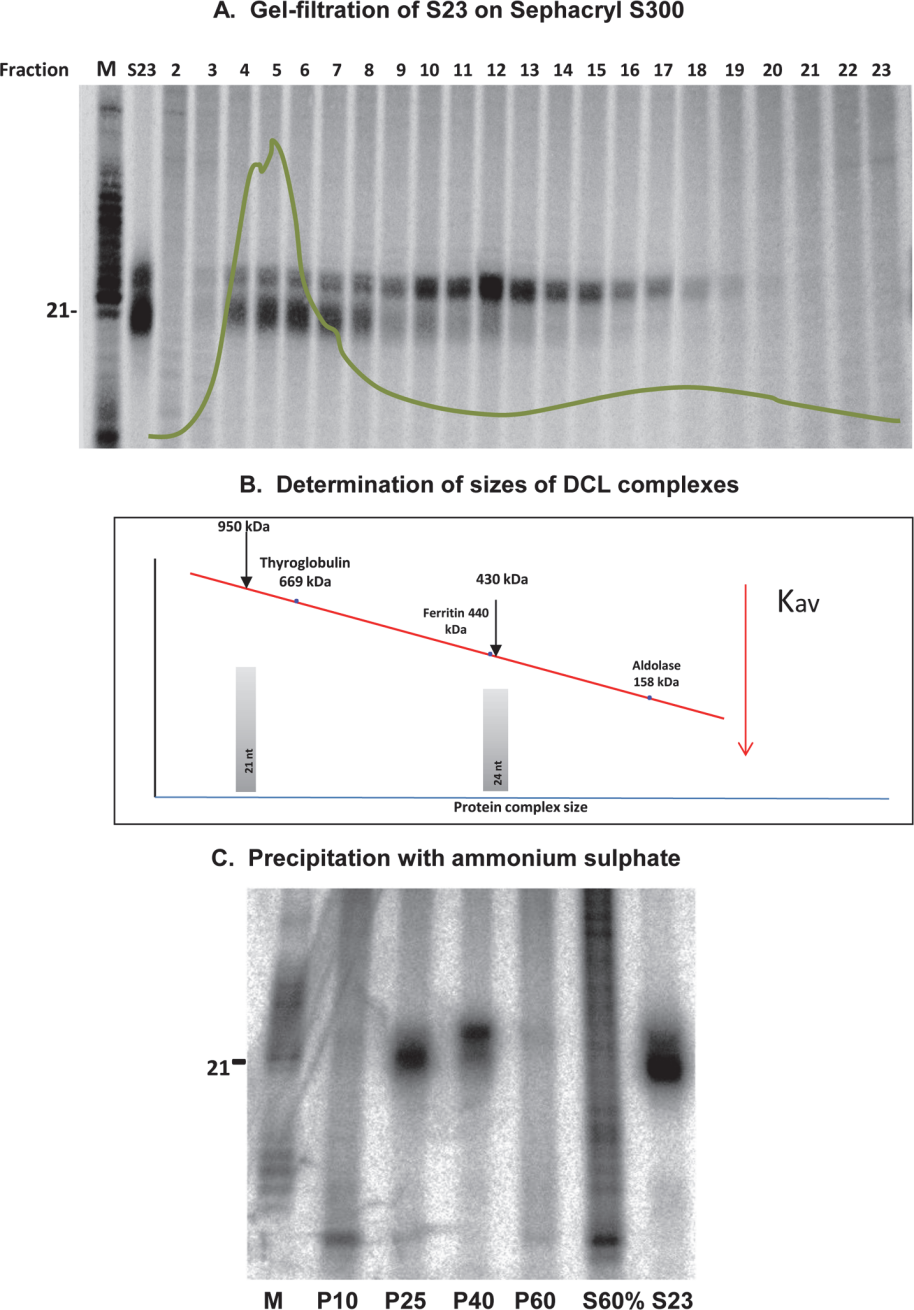
Purification of native DCLs from wheat germ extracts. **a**) Gel filtration analysis of crude wheat germ extract using Sephacryl S300. Fractions were collected and incubated with radiolabelled RNA substrates. The processed RNAs were precipitated with isopropanol, washed with 80% ethanol and dissolved in 10ul of loading dye (100% deionized formamide and 0.1% bromophenol blue) before separating on a 15% polyacrylamide gel. OD of the fractions was calculated separately and merged as green line with the gel picture. **b**) Determination of the sizes of DCL complexes. Three known marker proteins were used to draw a standard curve and sizes of DCL complexes was inferred using this standard curve. **c**) Ammonium sulfate precipitation to separate 24 and 21 nt generating activities. Please see methods for details. Processed RNAs were detected as described above.

Tang and coworkers [59,63] have shown that wheat germ contains predominantly two DCL activities, one that generates ∼21 nt small RNAs (siRNA generating activity, [59]) and the other which generates ∼24 nt small RNAs (DCL3 activity). We prepared a crude extract of wheat germ similar to Tang et al. [59] (S23) and used this extract for analysis of small RNA generating activities by incubating with radiolabelled dsRNA substrates (please see methods). No small RNAs were generated when the protein extract was incubated with ssRNA or if the ss or dsRNA was incubated alone (data not shown). In our conditions, majority of the activity derived from wheat germ, unlike previously reported, was a 21 nt RNA generating activity (henceforth DCL4 activity). Gel filtration analysis of this extract using Sephacryl S300 and subsequent fraction collection and incubation with radiolabeled RNA identified two activities similar to the activities described by Tang et al. [59] (Fig. 1A). Fractions 5 and 6 predominantly had DCL4 complex, while fractions 12 and 13 had exclusively 24 nt siRNA generating activity (DCL3 activity). The size of the protein complex that generates ∼21 nt sRNAs was a huge complex of around 950 kDa, a fraction smaller than ribosomes (Fig. 1A and B). This size of DCL4 generating activity is higher than the DCL1 complex reported by Qi et al. for *Arabidopsis* [55]. This increase in size might have resulted from two reasons. The wheat DCL4 complex has additional partners that are not common in DCL complex derived from *Arabidopsis*, or the difference in source tissue (cultured cells or inflorescence for *Arabidopsis* and germ for wheat). Interestingly, the ∼24 nt sRNA generating complex is much smaller at around 450 kDa (Fig. 1B) matching with the DCL3 complex size as reported earlier for *Arabidopsis* [55].

Although fractions 5 and 6 have majority of DCL4 activity, there is also approximately 10% of 24 nt sRNA generating DCL3 activity, thus prompting further purification of the protein complex. Ion exchange chromatography with mono-Q as well as SP and DEAE was not successful in completely removing the 24 nt RNA generating DCL3 activity from DCL4 activity (data not shown). This prompted us to perform additional steps of purification before subjecting the crude extract S23 to gel filtration.

A series of saturations with ammonium sulphate that is ideal in precipitating proteins was performed using S23 extract (Fig. 1C). The pellet formed either at 10% saturation and at 60% did not have any detectable DCL activities indicating that both the 21 and 24 generating activities precipitate within the range of 10% and 60%. We performed a stepwise precipitation and identified critical saturation points for individual DCL activities. At a saturation of 25%, almost pure 21 nt RNA generating activity precipitated (Fig. 1C) and this was subjected to gel filtration. Majority of 24 ntRNA generating activity precipitated at 40% saturation, but had a significant fraction of DCL4 activity. We used respective precipitated fractions for gel filtration using sephacryl S300. The DCL4 activity was easily fractionated, while the DCL3 activity with still a fraction of 21 nt generating activity (Fig. 1C) was now amenable for purification using sephacryl S300 resulting in fractions with DCL3 activity alone (S2A Fig.). The individual activities were further purified using ion exchange chromatography. The DCL4 was highly unstable leading to loss of activity during ion exchange chromatography with various techniques such as Q sepharose, Mono Q (anion exchangers), S, CM and SP (cation exchangers) and hydroxyapatite (data not shown). However, DCL3 activity was stable enough to resist ion exchange chromatography (S2B Fig.). A schematic representation of purifications of native DCL complexes from wheat germ has been shown in S1 Fig. Through these complex processes using ammonium sulfate precipitation followed by gel filtration and ion exchange, substantial amount of DCL4 and DCL3 activities were generated. Unlike the *Arabidopsis* extracts with such activities, wheat germ derived extracts were stable through freeze-thaw cycles and were amenable to study *in vitro* requirements for DCL activities.

### Requirements of salt for dicing activities

**Figure 2:**
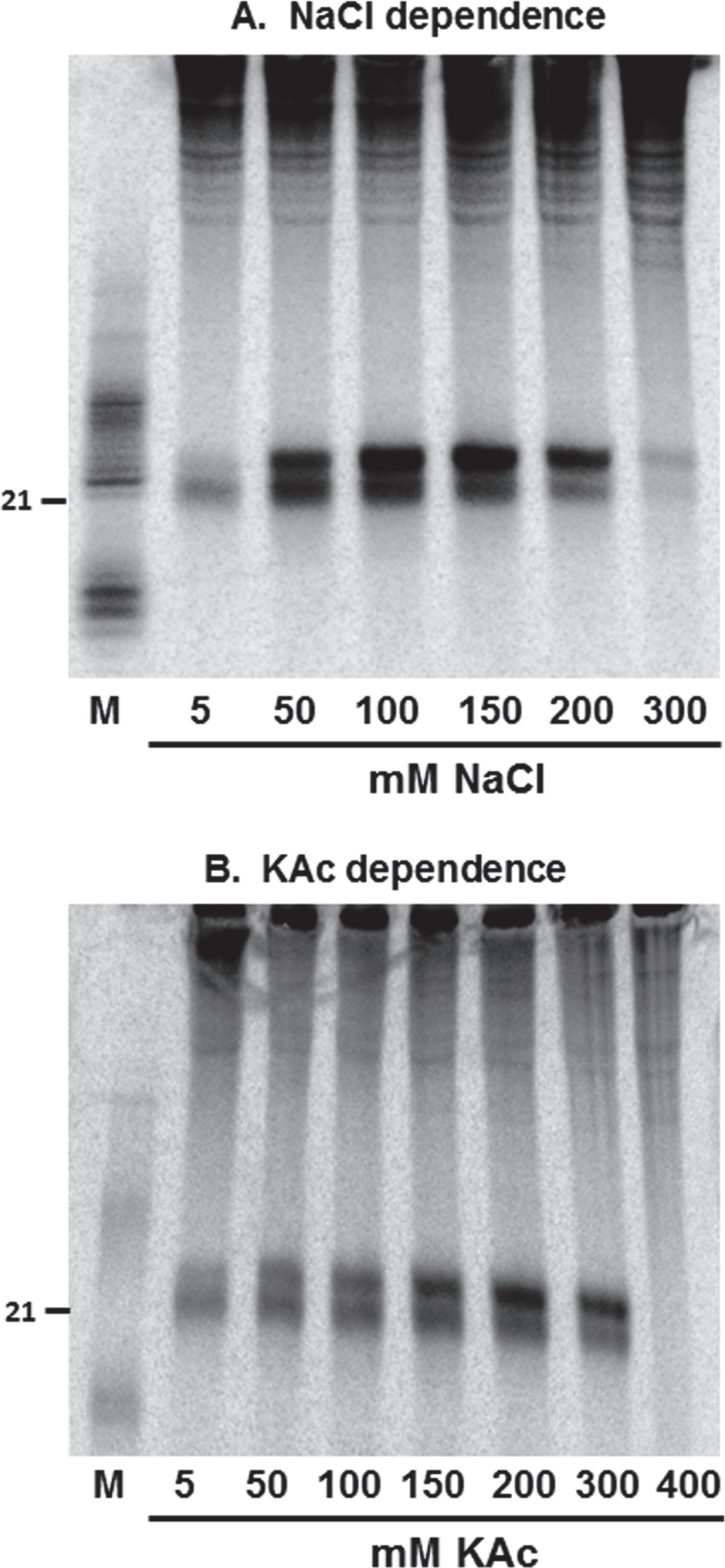
Requirements of salt for DCL activities. **a**) NaCl dependency of DCL activities. S23 extract was used to calculate optimal NaCl concentration required for DCL activities by providing labelled substrates. The extracts were first desalted by passing two times through sephadex G25 columns with buffers having 5 mM of the salt. Range of concentrations of NaCl used is shown. **b**) KAc dependency of DCL activities. Further details are similar to **a**.

It was shown previously that DCL activities require salt for their activity with an optimal range of 50–100 mM NaCl as reported for human Dicer [64] and plant DCL1 [58]. It is also well known that ribonuclease action depends on concentration of salts in the reaction [65]. We used crude extract to check the optimum concentration of NaCl and KAc for DCL activities, two commonly used salt activators during ribonuclease digestions. The reaction mix (please see Methods) was modified to have either KAc or NaCl at different concentrations. In these assays DCL4 complex was surprisingly active, albeit at lower levels, with minimum salt in the reaction mix (both NaCl and KAc), but peaked with NaCl concentration at 50–100 mM similar to what was described for human Dicer (Fig. 2, [64]). At the same time, DCL4 activity was almost unaltered at varying concentrations of KAc (Fig. 2B) indicating clearly that NaCl is the preferred salt for DCL4 activity. The DCL3 activity, however, was much more responsive to higher concentrations of NaCl, with a peak activity observed between 100 and 150 mM. DCL3 activity was also high at 200 mM of KAc unlike DCL4. The concentration of salt required for DCL4 activity matched that of DCL1 reported earlier [58], but DCL3 requirement is much higher than any other Dicer activity observed so far.

### Divalent cation dependence of native DCL activities

In *E. coli* RNase III, Mg2+ and Ca2+ act to stabilize complex formation of the enzyme with the bacteriophage T7 R1.1 RNA, which has a hairpin structure [66]. Binding of human Dicer, an RNAse III type enzyme, is not dependent on Mg2+ since Dicer has been shown to form stable complex with dsRNA even in the absence of Mg2+ [67]. However, it has been shown that human Dicer absolutely requires Mg2+ to be catalytically-active [64]. Other divalent cations like Mn2+ and Co2+ can substitute for Mg2+ in supporting the catalytic activity of Dicer [68] but activity of Dicer in the presence of other divalent cations has not been reported for human or for other Dicers from other species.

**Figure 3:**
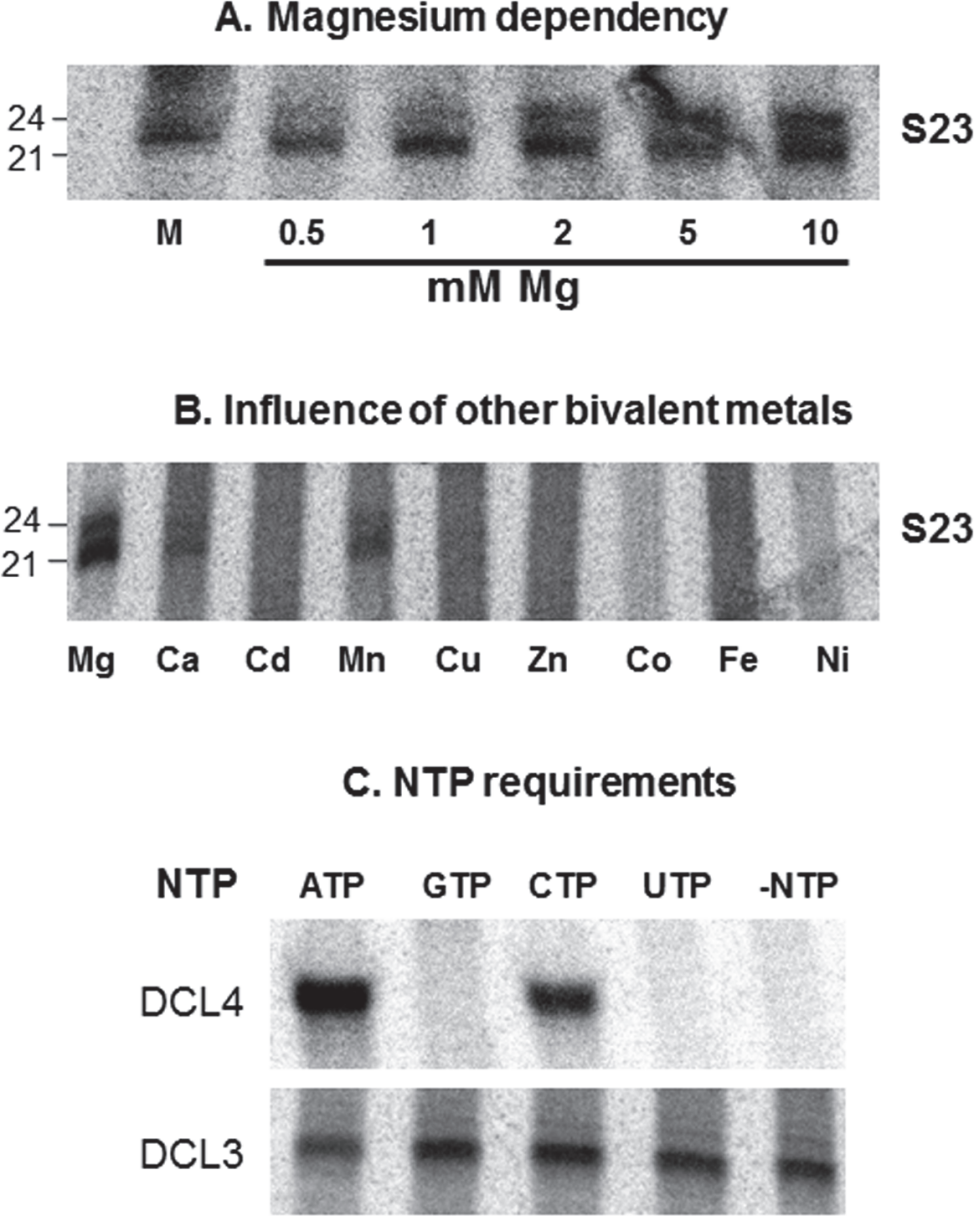
Bivalent metal and NTP requirements. **a**) Mg2+ dependency of DCL activities. S23 extract was used to calculate optimal Mg2+ concentration (mM) required for DCL activities by providing labelled substrates. **b**) Influence of other bivalent cations for DCL activities using S23 extract. All bivalent cations were supplied at 2 mM concentration. **c**) NTP requirements for DCL activities. The extracts were desalted to avoid NTPs present in the complex interfering with the data. Extracts were incubated with one of the NTP mentioned at 500 uM each. –NTP lane had extracts without addition of NTP. For more details, please refer Fig. 1 legend.

Similar requirement of divalent cations for plant DCLs has not been explored for native complexes, however, Mg2+ is absolutely required for *E. coli* expressed DCL1 to cleave a miRNA precursor [69]. In order to identify bivalent ion requirements for DCL cleavage in native complexes, we initially depleted the crude fraction S23 with metal ions by passing through G25 (GE healthcare) columns. Series of Mg2+ containing cleavage buffers were made and radiolabelled substrates were incubated with protein fractions. Effective concentration of Mg2+ best suited for DCL4 cleavage was identified as 1 to 2 mM (Fig. 3A). Surprisingly, DCL3 complex required slightly higher amount of Mg2+ at 10 mM. In plants, some divalent cations may inhibit DCL activities. The heavy metal cadmium (Cd2+) has been shown to inhibit multiple aspects of PTGS, likely reducing siRNA generation [70]. In order to understand if this is the case, we used several divalent ions, all at 2 mM concentration, and then performed cleavage assays. Not surprisingly, Mg2+ was the best ion, but the complexes were also active with Ca2+ and Mn2+. Irrespective of the activity (either DCL4 or DCL3), cleavage ability was inhibited in the presence of few metal ions including Cd2+ (Fig. 3B).

### DCL3 activity is independent of NTP supplement while DCL4 requires ATP and CTP

Human Dicer requires ATP for its activity [64]. Recombinant DCL1 from *Arabidopsis* also required ATP for activity but GTP also promoted DCL activity albeit at lower levels when compared to ATP [69]. Wheat germ DCL4 activities required ATP, while the efficiency of cleavage with other NTPs was not evaluated [63]. In order to understand the NTP requirements for the DCL4 and DCL3 activities of wheat germ, cleavage assays in the presence of optimal concentrations of ATP, CTP, UTP and GTP was performed (Fig. 3C). The DCL4 activity required ATP and surprisingly to some extent CTP (Fig. 3C, upper panel), while 24 nt processing was independent of NTP supplement (Fig. 3C, lower panel). Our results are in line with that of Tang et al. [63] who show that some DCL3-like activity was still functional in the absence of ATP.

### Temperature and pH requirements

**Figure 4:**
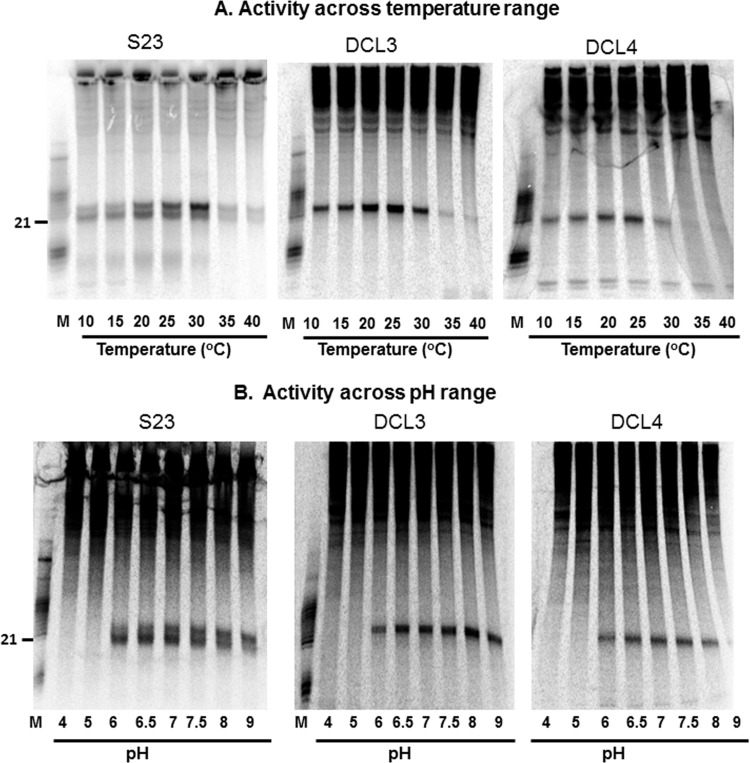
pH and temperature requirements. **a**) Activity of DCL complexes across temperature range using S23 and individual DCL complexes. Range of temperature used is mentioned. Please refer Fig. 1 legend for other details. **b**) Activity of DCL complexes across a pH range using DCL activities.

Most enzymatic reactions require specific pH and temperature and DCLs must have a specific requirement. It has been widely shown that plant PTGS is inhibited at low temperatures [71]. However, there are few experiments carried out to test optimal temperature for Dicer activity. Zhang et al. [64] showed that Proteinase K treatment somehow enhanced the activity even as low as 4°C temperature. In their experiment, untreated Dicer was most active at 37°C. Similarly, effect of pH has not been studied widely. In order to understand the optimum temperature at which native DCLs are active, we tested the ability of both 21 and 24 nt producing activities under varying temperatures between 10°C and 40°C. There was a dramatic difference in the optimal temperatures at which these activities peaked. DCL3 activity peaked at 30°C as seen from higher abundance of 24 nt siRNAs generated (Fig. 4A), while DCL4 activity peaked between 20–25°C. The activities in crude extracts showed a minor difference for optimal activity when compared to individual fractionated activities. This change could have come from additional factors present in crude extracts that may have been removed during purification. The DCL4 activity was also efficient at very low temperatures of 10°C, but was inactive at 35°C. The differences between the two activities tested may have resulted from multiple factors, including their localization, nature of partners and/ or stability. Nevertheless, this difference may contribute to their functional differences.

Similarly, at varying pH ranging between 4 and 9, crude extract as well as individual activities were incubated with radiolabelled substrate (Fig. 4B). The DCL4 cleavage peaked at pH7 and was inhibited at alkaline pH. The 24 nt cleavage activity of DCL3 was active at alkaline pH with a peak activity at pH8. This can be best compared with S23 that has both activities-the window of 24 nt activity is more towards alkaline pH as opposed to 21 nt activity. Crude extract again exhibited differences for pH requirement when compared to fractionated activities.

### RNA structural determinants effecting processing by native DCL complexes

**Figure 5:**
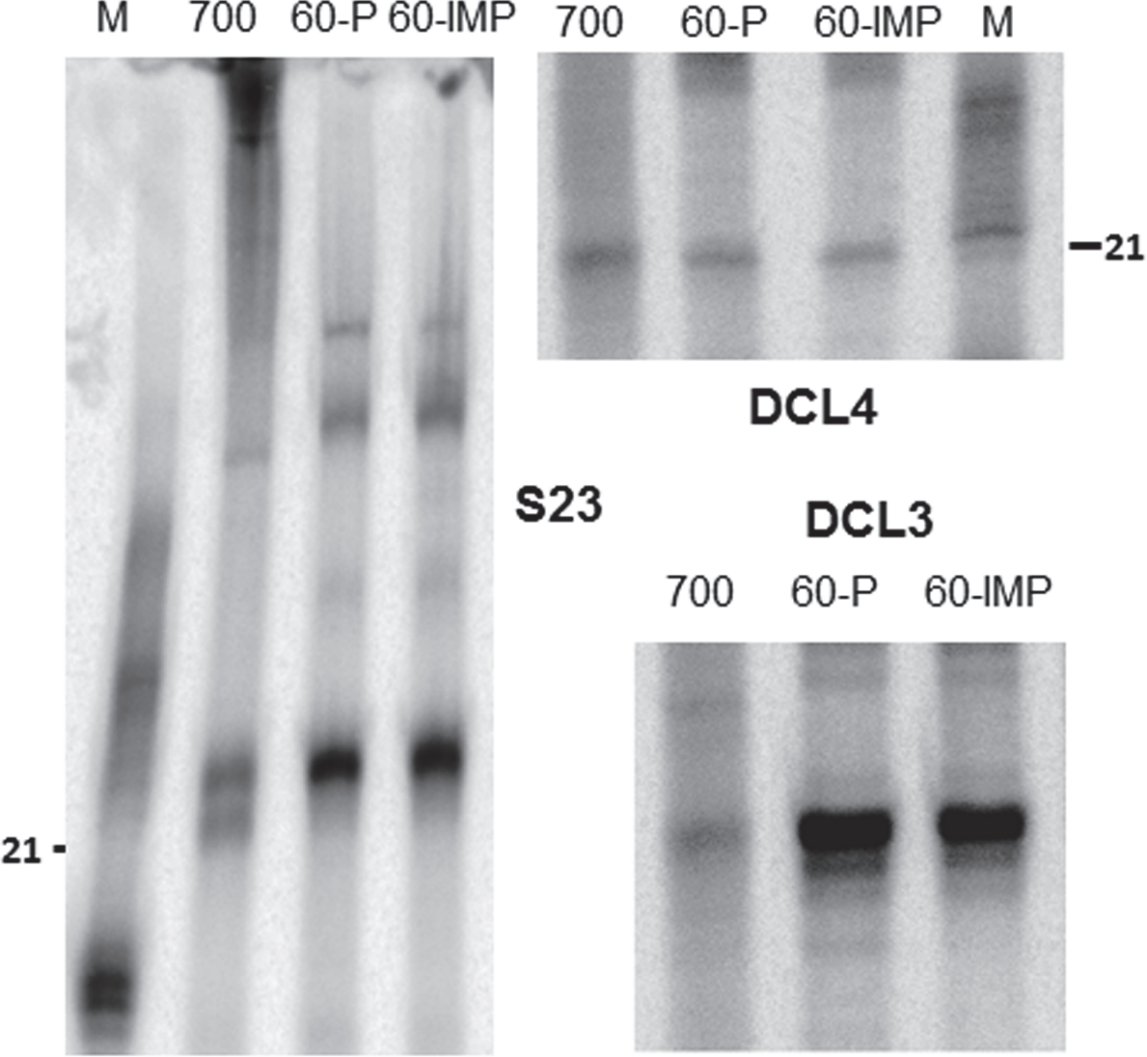
Substrate processing abilities of DCL activities. Perfectly complementary (P) or imperfectly complementary (IMP; having a mismatch every 4^th^ base) substrates of the mentioned length were incubated with DCL activities to analyze their processing abilities. Note that 700 bp length substrate is perfectly complementary (Please see methods).

A highly structured RNA is the source of miRNAs among plants. Sources of siRNAs are thought to be completely complementary RNAs of substantial length. In order to understand the substrate processing abilities of native DCL4 and DCL3 complexes, we incubated both perfectly complementary and imperfectly complementary RNAs with native complexes (Fig. 5). A labeled 700 bp RNA that can form secondary structures was processed efficiently to produce 21 nt and 24 nt siRNAs in the crude extract containing DCL3 and DCL4 complexes. It is surprising to note that this substrate although has low complementarity, could still be substrate for both these activities thought to be requiring completely complementary dsRNA as substrate. However, viruses that produce structured RNAs have been shown to be targeted by multiple DCLs during virus infection [72,73].

Length of the substrate may also affect processivity of Dicers and DCLs [64]. Incubation of DCL activities with a smaller 60 bp dsRNA of both completely complementarity and incompletely complementary dsRNA, led to production of abundant siRNAs. DCL3 activity was much more active in dicing both completely complementary as well as incompletely complementary substrates of 60 bp length when compared to the DCL4 activity. This is surprising due to the fact that DCL4 is supposed to be active in cleaving and degrading RNA [57,74,75] at a faster rate than many endonucleases, but has lower efficiency than DCL3, nature of its ability not reported in literature yet.

## Discussion

DCL proteins are RNAse III type nucleases that cleave structured RNA species to produce small RNAs. These small RNAs are incorporated into effectors called Argonautes to target RNAs that have complementarity with the small RNAs. Genetic data and observations made using virus infected plants showed that DCLs have specificity for their substrates. Genetic data also showed, based on some of the signatures of DCL activities in virus infected or stressed plants, that certain environmental factors influence activities of DCLs. Despite RNA silencing being discovered in plants, biochemical analyses of silencing players in plants such as *Arabidopsis* have met with little success, especially because the plant DCL complexes are huge, low abundant, have many partners and their extracts are unstable [76]. Thus, most of the mechanistic understanding of plant silencing came from mutation studies. However, unlike animal systems, plants have diversity in DCL genes (4 variants in Arabidopsis) and AGOs (10 in Arabidopsis), with partially redundant functions, thus conclusive mechanistic understanding of their functions from studies with mutants remains a challenge. Biochemical characterization of dicers in animals, on the other hand, turned out to be simple as the heterologously expressed Dicer could function independent of partners [64]. However, heterologous expression of plant Dicers alone could not cleave labeled RNAs indicating that partners of unknown nature are absolutely required for DCL activities in plants (data not shown). In addition, most AGOs and DCLs are abundant in specific tissues such as those involved in reproduction and using these tissues to isolate native complexes has not been trivial.

We used wheat germ derived purified native complexes of DCLs unlike most reported studies on Dicer activities and requirements. Our observations are likely applicable to other monocots and some dicots as well. DCL complexes used here are likely to have all co-factors and accessory factors necessary for their activity. This system is highly advantageous when compared to recombinant proteins expressed in artificial heterologous systems for at least two reasons. The protein behavior of wheat germ derived complexes is likely similar to *in vivo* conditions, in contrast to the behavior of recombinant proteins. Indeed, small RNA profiles observed in our assays match well with those found in plants under normal conditions as well as during stresses, such as temperature and cadmium stress. Complexes reconstituted from individual components may behave differently than the natural complete ones due to incomplete reconstitution or incomplete modification of the components and thus may provide erroneous results. For example, plant DCL1 expressed in *E. coli* can cleave a long RNA but the small RNAs generated from this activity did not have signatures of typical miRNAs indicating that isolated DCLs possess the ability to process various RNA substrates but are unlikely to match the precision of the complete DCL1 complex. However, when HYL1 (DRB1), identified as a partner required for accurate processing of long RNA, was provided, the activity became specific and matched *in vivo* results. We believe that our purified complexes match *in vivo* conditions perfectly. Our method using purified complexes is slightly better using crude extracts because crude extracts contain various additional proteins, unknown co-factors that may associate with DCLs to influence the biochemical requirements and substrate specificities. It has been shown for example that individual DRBs that are essential for distinctive DCL activities may also partner with other DCLs. Such a scenario is easily possible in crude extracts since there are opportunities to have unlimited number and amounts of proteins that may be natural or conditional partners.

In our assays we found some properties of DCL complexes correlating well with genetic and whole-genome analysis. These include restriction of optimal temperature for efficient silencing of viruses and production of endogenous siRNAs. In our assays, the activity of DCL4, a major antiviral protein, as well as a participant of transgene PTGS, is restricted to optimal conditions. It has negligible activity at 30°C and above. Indeed it has been shown that by moving *Arabidopsis* plants from 22°C to 30°C, transgene-induced PTGS becomes severely inhibited [77]. Concurrently, production of *trans*-acting siRNAs produced by DCL4 also get inhibited at 30°C [77]. Our data also correlate well with other reports where low temperatures, e.g., 15°C, inhibit transgene and virus-induced silencing [71]. In our assays too, optimal temperature for activity of DCL4 and DCL3 are much higher than 15°C. For all parameters except for NTP requirements, DCL3 has a more restricted activity window than DCL4. The implications for this restricted requirements of DCL3 has not been fully understood. However, it is important to consider that DCL3 is predominantly a nuclear protein and thus the restricted range could have been imposed due to its restricted localization.

Substrate requirements for DCL3 in our assays have been surprising. DCL3 processes dsRNA substrates that have been thought to be completely complementary [78]. However, in our assays DCL3 cleaved imperfectly complementary dsRNA as efficiently as completely complementary dsRNA. However, the efficiency of cleaving long dsRNA was lower than for completely complementary and incompletely complementary short substrates of 60 bp.

Further understanding of activities and structures of DCLs should elucidate how plant DCLs work in a more precise manner. Wheat germ system in the context of such details and identifying partner proteins need backing from genome sequence data, that is now available [79].

## Supporting Information

S1 Fig Scheme of DCL purification from wheat germ. (TIF)

S2 Fig Further purification of Ammonium sulfate pellets to obtain pure DCL4, DCL3 complexes. **a**) Gel filtration of 40% saturated ammonium sulfate pellet to remove unwanted proteins. Assay was carried out to purify DCL3 from DCL4 background. See methods for more details. **b**) Q-Sepharose chromatography to purify DCL3 complex from DCL4 background. Concentration of NaCl used for elution is mentioned.
(TIF)
