# Supplementary figures and images for: Biochemical Requirements for Two Dicer-Like Activities from Wheat Germ
Source: PLoS One. 2015 Jan 23;10(1):e0116736. doi: 10.1371/journal.pone.0116736 (PMC4304710; doi:10.1371/journal.pone.0116736)

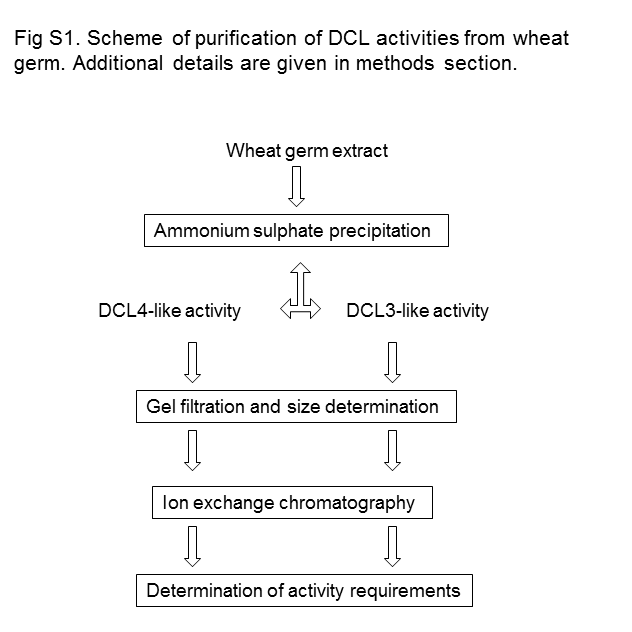

Supplement: S1 Fig — (TIF) [file pone.0116736.s001.tif]

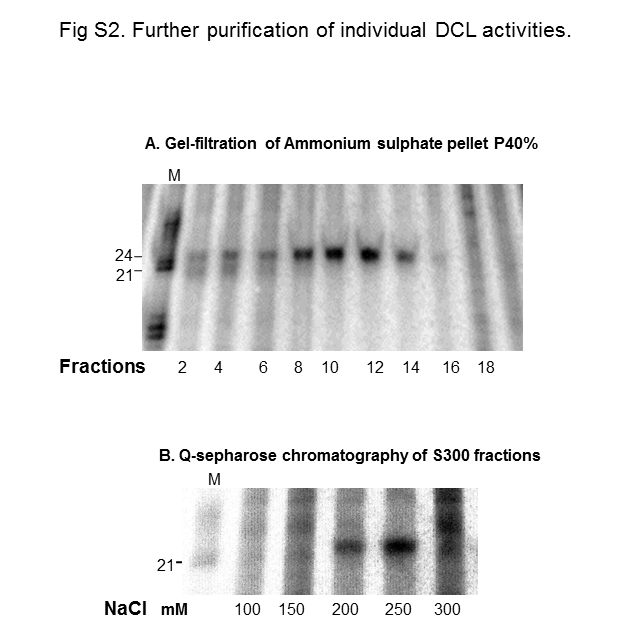

Supplement: S2 Fig — a) Gel filtration of 40% saturated ammonium sulfate pellet to remove unwanted proteins. Assay was carried out to purify DCL3 from DCL4 background. See methods for more details. b) Q-Sepharose chromatography to purify DCL3 complex from DCL4 background. Concentration of NaCl used for elution is mentioned. (TIF) [file pone.0116736.s002.tif]
